# Assessment of HIF-1α expression and release following endothelial injury in-vitro and in-vivo

**DOI:** 10.1186/s10020-018-0026-5

**Published:** 2018-05-16

**Authors:** Lamia Heikal, Pietro Ghezzi, Manuela Mengozzi, Gordon Ferns

**Affiliations:** 1 Brighton and Sussex Medical School Department of Clinical and experimental investigation, University of Sussex, Falmer East Sussex, Brighton, BN1 9PS UK; 2 Brighton and Sussex Medical School Department of Medical Education, Mayfield House, Falmer East Sussex, Brighton, BN1 9PH UK

**Keywords:** HIF-1α, Injury, Endothelial cells, ELISA, Angioplasty

## Abstract

**Background:**

Endothelial injury is an early and enduring feature of cardiovascular disease. Inflammation and hypoxia may be responsible for this, and are often associated with the up-regulation of several transcriptional factors that include Hypoxia Inducible Factor-1 (HIF-1). Although it has been reported that HIF-1α is detectable in plasma, it is known to be unstable. Our aim was to optimize an assay for HIF-1α to be applied to in vitro and in vivo applications, and to use this assay to assess the release kinetics of HIF-1α following endothelial injury.

**Methods:**

An ELISA for the measurement of HIF-1α in cell-culture medium and plasma was optimized, and the assay was used to determine the best conditions for sample collection and storage. The results of the ELISA were validated using Western blotting and immunohistochemistry (IHC). In vitro, a standardized injury was produced in a monolayer of rat aortic endothelial cells (RAECs) and intracellular HIF-1α was measured at intervals over 24 h. In vivo, a rat angioplasty model was used. The right carotid artery was injured using a 2F Fogarty balloon catheter. HIF-1α was measured in the plasma and in the arterial tissue (0, 1, 2, 3 and 5 days post injury).

**Results:**

The HIF-1α ELISA had a limit of detection of 2.7 pg/mL and was linear up to 1000 pg/ mL. Between and within-assay, the coefficient of variation values were less than 15%. HIF-1α was unstable in cell lysates and plasma, and it was necessary to add a protease inhibitor immediately after collection, and to store samples at -80 °C prior to analysis. The dynamics of HIF-1α release were different for the in vitro and in vivo models. In vitro, HIF-1α reached maximum concentrations approximately 2 h post injury, whereas peak values in plasma and tissues occurred approximately 2 days post injury, in the balloon injury model.

**Conclusion:**

HIF-1α can be measured in plasma, but this requires careful sample collection and storage. The carotid artery balloon injury model is associated with the transient release of HIF-1α into the circulation that probably reflects the hypoxia induced in the artery wall.

**Electronic supplementary material:**

The online version of this article (10.1186/s10020-018-0026-5) contains supplementary material, which is available to authorized users.

## Background

Endothelial injury is an early and enduring feature of cardiovascular disease. Inflammation and hypoxia may be partially responsible for this, and are often associated with the up regulation of transcriptional factors that include hypoxia-inducible factor-1 (HIF-1) (Ferns and Heikal 2017).

HIF-1 is a hetero-dimeric transcription factor that consists of an oxygen-regulated α- subunit and a constitutively expressed ß-subunit (Chu and Jones 2016). HIF-1β is expressed in the nucleus and its activity is unaffected by hypoxia, whereas the HIF-1α subunit has a short half-life (5 min) and its stability is regulated by oxygen tension (Gao et al. 2012). Intracellular HIF-1α is unstable under normoxic conditions, being rapidly degraded by prolyl hydroxylase enzymes (Semenza 2014). These enzymes are inactivated under low oxygen levels, explaining why HIF-1α is induced by hypoxia. Some small molecules such as dimethyloxalylglycine (DMOG) can stabilize HIF levels under normoxic conditions by inhibiting prolyl hydroxylase (Zhang et al. 2016). Three distinct HIF-α subunits have been identified (HIF-1α, HIF-2α and HIF-3α) that have a tissue-specific pattern of expression (Al Okail 2010). HIF-1α and HIF 2α are similar but not identical. HIF-1α is expressed in response to acute hypoxia, whilst HIF-2α is related to chronic responses (Loboda et al. 2012). HIF-1 drives vascular endothelial growth factor (VEGF) expression and angiogenesis while HIF-2 production blocks angiogenesis by inducing the expression of the soluble VEGF receptor-1, sequestering biologically active, free VEGF. It is possible that HIF-2α evolved to regulate the VEGF response to hypoxia and the resultant development of the vascular network (Eubank and Marsh 2011; Eubank et al. 2011; Loboda et al. 2012). Therefore, HIF-1 is a key transcription factor in the adaptive responses to low oxygen (Lambert et al. 2010). HIF-1 is involved in the cellular adaptation to injury, inflammation, infection and cancer (Eubank and Marsh 2011). HIF is also induced by inflammatory cytokines including tumour necrosis factor alpha (TNF-α) and interleukin-1β (IL-1β), that increase the accumulation and transcriptional activity of HIF-1α (Gao et al. 2012).

HIF-1 also regulates a variety of other important cellular responses affecting a spectrum of protective/reparative processes including angiogenesis, cell proliferation and survival (Ferns and Heikal 2017; Lambert et al. 2010). It promotes the transcription of more than 100 target genes including several angiogenic factors, apart from VEGF, nitric oxide synthase (the inducible and the endothelial forms), platelet-derived growth factor, and erythropoietin (EPO) (Gao et al. 2012; Natarajan et al. 2007).

Several semi-quantitative methods have been used to estimate HIF-1α levels, for example Western blotting and immunocytochemistry (Karshovska et al. 2007; Srinivasan and Dunn 2011). HIF-1α levels have also been assessed indirectly by quantifying mRNA expression, or by measuring downstream target genes such as VEGF and EPO (Formento et al. 2005; Park et al. 2014).

Due to the need for a more precise method for HIF-1α quantification in biological samples, some quantitative methods have also been developed, that include the enzyme linked immunosorbent assay (ELISA) (Formento et al. 2005). Although it has been reported that HIF-1α is detectable in plasma, (He et al. 2016a) it is unstable and is rapidly degraded under normal oxygen tensions, as discussed above (Park et al. 2014). As a transcription factor, it is normally located within the cell, and there has also been some doubt about its release into plasma (Ferns and Heikal 2017).

Plasma HIF-1α may potentially represent a biomarker of vascular wall hypoxia, or the extent of atherosclerosis. It may also reflect the severity of hypoxia associated with other pathological conditions including some tumours. Hence, there are a number of possible clinical applications for a plasma HIF-1α assay.

Our aim was to optimize an assay for HIF-1α to be applied to in vitro and in vivo studies, and to establish the conditions for sample collection and storage. We then aimed to use the assay to assess the release kinetics of HIF-1α following endothelial injury in vitro and in vivo*.*

## Methods

All chemicals were from Sigma Aldrich (Dorset, UK), unless otherwise stated.

### Cell culture

Rat aortic endothelial cells (RAECs) were isolated from the aortae of Sprague Dawley rats (male, 2 weeks old) and used between passages 2–5 (Kobayashi et al. 2005). The cells were cultured in Endothelial cell growth medium supplemented with 10% fetal bovine serum (FBS) and Penicillin/Streptomycin (final concentration 100 IU/mL), and were cultured, prior to our experiments, at 37 °C in a humidified atmosphere containing 5% CO_2_ and 21% O_2_. When indicated, experiments under hypoxic conditions were performed under 1% O_2_, 5% CO_2_ and 94% N_2_.

### Effect of storage on the HIF-1α stability

Storage time and temperature, and the effects of adding protease inhibitors to the experimental samples were investigated to determine the optimum storage conditions for HIF-1α measurements.

For the effect of temperature, aliquots of samples were taken and maintained at different temperatures (− 80, − 20, 4 and 37 °C). HIF-1α concentrations were determined after 24 h storage using the ELISA as described below.

To determine the effect of storage duration on HIF-1α concentrations, fresh samples were divided into 3 aliquots, and HIF-1α was measured immediately, or after 1 and 2 weeks. Aliquots were stored at -80 °C until analysis.

To assess the requirement for the addition of a protease inhibitor, aliquots from a set of fresh samples were treated with the protease inhibitor, aprotinin (300 nM) (Mosher et al. 2014). These samples were stored for 1 or 2 weeks at -80 °C until they were analyzed.

Linearity, limit of detection and limit of quantification of the assay were determined. Assay precision was assessed by measuring intra and inter-plate replicates with appropriate samples and HIF-1α standards.

### Quantification of HIF-1α after administration of a HIF-1α inducer

Cells were seeded into 24 well plates with a seeding density of 1 × 10^5^ cell/mL and cultured until 80% confluent. The HIF-1α inducer; DMOG was added at different concentrations ranging from 0 to 500 μM and incubated for 24 h at 21% O_2_. Cells incubated in hypoxic conditions (1%O_2_) were used as a positive control for HIF-1α induction. Cells were then lysed and HIF-1α was measured using the ELISA method. In another set of experiments, cells were similarly treated with different concentrations of DMOG, lysed, and the down-stream effector, VEGF gene expression was measured.

### Scratch assay in vitro injury model

The “scratch assay” is a model of cell/ wound injury, and has been described previously (Heikal et al. 2015). Briefly, a reproducible scratch was produced in the endothelial monolayer, and the cells were then incubated in room air (21% O_2_) in a 5% CO_2_ incubator. Cells were then lysed and intracellular HIF-1α assessed. This was evaluated over a period of 24 h using the ELISA.

### Animal studies

Male Sprague Dawley rats (450 g) were used. All animal experiments were performed under UK Home Office approval according to the Animals Scientific Procedures Act, 1986 and subsequent revisions, and conformed to the Guide for the Care and Use of Laboratory Animals published by the National Institutes of Health (NIH Publication No. 85–23, revised 1996). Studies were designed and conducted in accordance with the ARRIVE guidelines (Kilkenny et al. 2010). Rats were acclimatized in home cages for 1 week prior to experimentation with ad libitum access to food and water and on a 12h light-dark cycle.

### Quantification of HIF-1α post DMOG administration

Rats were anesthetized using 2% isoflurane and placed in a supine position. The right carotid artery was exposed and DMOG was applied locally at different concentrations (0–7.5 mg/Kg) on the artery using pluronic gel as the vehicle (30%*w*/w). Three rats were used for each concentration of DMOG. After 24 h, blood was collected from a tail vein, and HIF-1α was measured by ELISA.

Treated animals were culled after 24 h and both the right (treated) and left (untreated) carotid arteries were isolated and snap frozen for PCR analysis.

### Angioplasty in vivo injury model

Injury was induced in the rat’s carotid artery using a balloon catheter (for angioplasty) as previously described (Tulis 2007). Briefly, rats (450 g) were anesthetized using 2% isoflurane and placed in a supine position. The right carotid artery was exposed and a 2F Fogarty catheter was used to cause injury. The catheter was inserted into the common carotid artery via the external carotid to cause complete removal of the vascular endothelium from the common carotid artery down to its junction with the aortic arch.

Blood was collected from a tail vein at 0, 1, 2 and 5 days after injury into EDTA containing sample tubes. Plasma was separated and used for the measurement of HIF-1α using ELISA.

At the end of the experiment, rats were culled. Both the right (treated) and left (untreated) carotid arteries were isolated and snap frozen for PCR analysis.

In another set of experiments culled animals were perfused fixed with PBS followed by 4% paraformaldehyde. Both the right (treated) and left (untreated) carotid arteries were isolated and kept in 4% paraformaldehyde at room temperature for 1 h. Fixed arteries were then placed in a 30% sucrose solution and left overnight after which they were snap frozen in OCT blocks for cryo-sectioning.

### HIF-1α enzyme linked immunosorbent assay (ELISA)

HIF-1α was measured using a commercial ELISA kit (R&D systems/ Biotechne, UK) following the manufacturers’ instructions.

- Standard calibration curves were obtained using HIF-1α protein standards, allowing the limit of detection, limit of quantification and coefficient of variation to be determined.
- In vitro *samples:* Endothelial cells were lysed in 80 μL lysis buffer (25 mmol/L Tris HCl pH 7. 6, 0.1% SDS, 1% deoxycholate, 1% NP40, 0.5 mol/L EDTA, 40 mmol/L EGTA and protease inhibitors). Lysates were then centrifuged at 11000 *g* for 15 min at 4 °C and the supernatant was collected. Protein concentrations were quantified using a BCA reagent kit (Pierce Biotechnology). Results were expressed as pg/ mg protein.
- *Rat plasma:* Blood was collected from the tail vein of each animal and placed into tubes containing EDTA as an anti-coagulant. Blood was kept on ice and centrifuged to separate the plasma, to which a protease inhibitor was immediately added. HIF-1 was then quantified.

### Real time qPCR

The gene expression of vascular endothelial growth factor (VEGF was analysed by quantitative PCR (qPCR). Cells were lysed using TRIzol (Invitrogen, Life Technologies) and RNA was then extracted and purified. RNA quality and concentration were determined using a NanoDrop ND-1000 (NanoDrop Technologies). Reverse transcription and real-time quantitative PCR (qPCR) were carried out on RNA samples for VEGF and β2-microglobulin (a housekeeping gene not affected by changes in oxygen levels), using TaqMan gene expression assays (Applied Biosystems/Life Technologies). For gene expression quantification, the comparative threshold cycle (ΔΔCt) method was used following the manufacturer’s instructions. Results were normalized to β2-microglobulin expression and expressed as arbitrary units using one of the normoxic untreated samples as a calibrator.

For the analysis of rat tissue, RNA was extracted and purified from the frozen artery sections using TRIzol (Invitrogen, Life Technologies). RNA quality and concentrations were determined using a NanoDrop ND-1000 (NanoDrop Technologies). Reverse transcription and real-time quantitative PCR (qPCR) were carried out on RNA samples for VEGF and β2-microglobulin as described earlier.

### Western blotting

Western blotting was used to verify the presence of HIF-1α in some samples. Cells were lysed and the protein content of the lysate quantified as previously described (Heikal et al. 2015). Thirty μg of cellular proteins were separated on a 10% SDS-polyacrylamide gel electrophoresis and transferred onto a nitrocellulose membrane (Amersham/ GE Healthcare Life Sciences, Little Chalfont, Buckinghamshire, UK). After blocking with 5% skimmed milk (for HIF-1α detection) or 5% bovine serum albumin; BSA (for GAPDH detection) for 1 h, membranes were incubated with the appropriate primary antibody overnight, followed by HRP-conjugated secondary antibodies for 1 h at room temperature. HIF-1α and GAPDH (loading control) were detected using rabbit anti-HIF-1α (NB100-479, Novus biologicals, UK) and rabbit anti-GAPDH (14C10, Cell Signaling Technology, UK) at 1:500 and 1:1000 respectively and an anti-rabbit secondary antibody (A0545, Sigma Aldrich, UK) at 1:20,000 dilution. Protein bands were visualized by exposing membranes developed with the ECL reagent (Amersham/ GE Healthcare Life Sciences) to chemiluminescence film (Hyperfilm ECL, Amersham/ GE Healthcare Life Sciences). Bands were quantified using Image J software.

### Immunohistochemistry

Snap frozen carotid arteries were embedded in OCT and were cryo-sectioned (5 μm in thickness) and sections placed onto silane-coated slides. Sections were then washed with PBS and blocked using 10% donkey serum and 0.3% Triton X-100 for 1 h a room temperature, they were incubated with mouse anti-HIF-1α at 1:50 dilution in 1% donkey serum at 4 °C overnight. Donkey anti-mouse IgG-FITC (sc-2099, Santa Cruz Biotechnology, Germany) (1:100 dilution each) was the secondary antibodies used to detect HIF-1 (green stain). For comparing HIF-1α expression in different sections, instrument (fluorescence microscope) settings were kept constant for all sections using uninjured arteries as the control. In another set of experiments, cryo-sliced carotid artery sections were used to visualize endothelial cells (ECs) and smooth muscle cells post injury. Arteries were stained with rabbit anti-CD31 (NB100-2284, Novus biologicals, UK) at 1:50 dilution and mouse anti-alpha smooth muscle actin at  1:50 dilution (NB2-33006, Novus biologicals, UK) followed by Alexafluor 647 conjugated anti-rabbit secondary antibody (red stain) and FITC-conjugated anti-mouse secondary antibody (green stain) respectively at 1:200 dilution each. Cellular nuclei were stained with Prolong® Gold anti-fade reagent with DAPI (P36941, Life technologies, UK). Images were obtained using a fluorescence microscope (Leica CTR 5000).

### Statistical analysis

All data were analyzed using Graph Pad Prism 4 software. Differences in treatment were tested for significance using one-way analysis of variance (ANOVA) followed by a Bonferroni correction for multiple comparisons post-hoc test.

## Results

### Reliability of the ELISA assay for the quantification of HIF-1α in biological samples

**Fig. 1:**
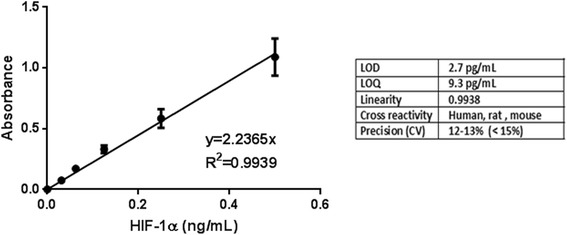
Calibration curve for standard HIF-1α αprotein at concentration range (0–500 ng/mL) using ELISA assay for protein quantification. The calibration curve was used to determine linearity, the limit of detection and quantification as well as inter and intra variability of the assay. Each point represents the mean ± SEM of 6 independent experiments

Figure 1 shows the results of a typical standard curve using standard recombinant HIF-1α. The assay was linear between 0 and 1000 pg/ mL (*r* = 0.99). The method was sensitive, with a detection limit of 2.7 pg/ mL. The ELISA had an intra and inter-assay coefficient of variation of < 15% and an accuracy of 99.4% ± 8.3%.

**Fig. 2:**
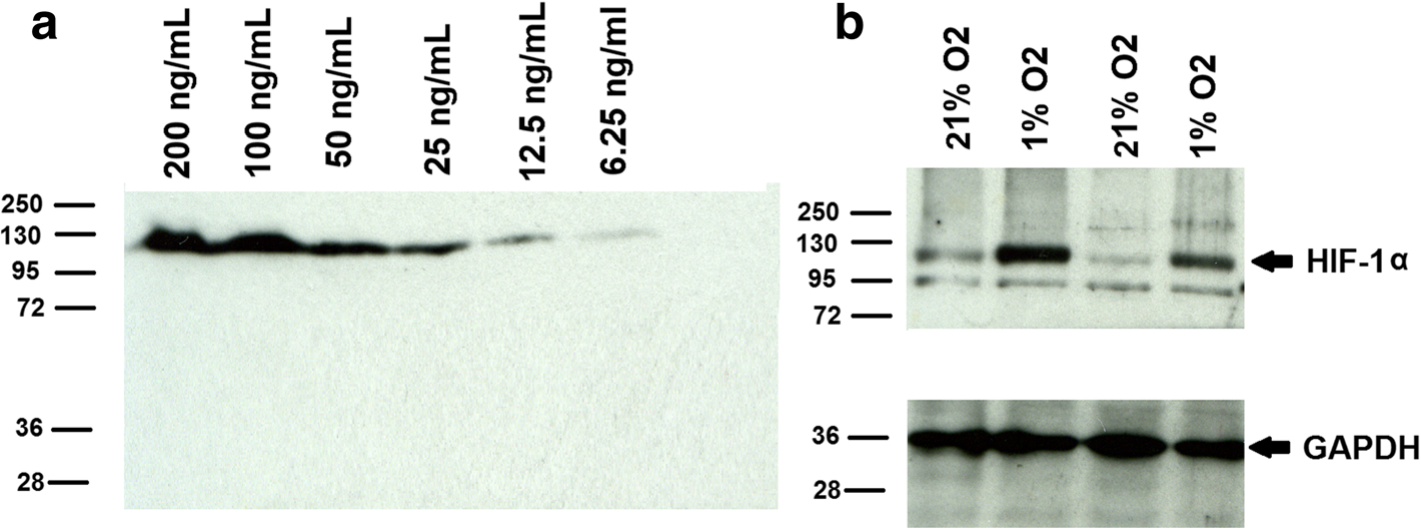
Western blot analysis of HIF-1α. **a** Different concentrations of HIF-1α standard protein and **b** cell lysates from the cells cultured in 21% or 1% O_2_. HIF-1α standards and in cell lysates showed a band at a molecular weight of approximately 130 kDa. GAPDH (loading control) showed a band at a molecular weight of approximately 36 kDa. Numbers on the left indicate the migration of molecular weight standards

The results of the HIF-1α measurement using the ELISA assay were compared with the findings using the conventional semi-quantitative assay; Western blot. The Western blot in Fig. 2a shows a specific band for the recombinant HIF-1α standard protein at a molecular weight of approximately130 kDa with a band intensity that was concentration dependent and detectable in the range used (6–200 ng/mL). When cell lysates were analyzed by Western blot, we could detect a specific band for HIF-1α at the molecular weight of 130 kDa in addition to a faint degradation band at 80 kDa. (Lidgren et al. 2005; Srinivasan and Dunn 2011) A stronger signal of the higher molecular weight band was detected in cells cultured for 24 in hypoxia. GAPDH was used as a loading control showing a band in each cell lysate at molecular weight 40 kDa.

### Temperature, storage time and presence of protease inhibitors are critical for the stability of HIF-1α

**Fig. 3:**
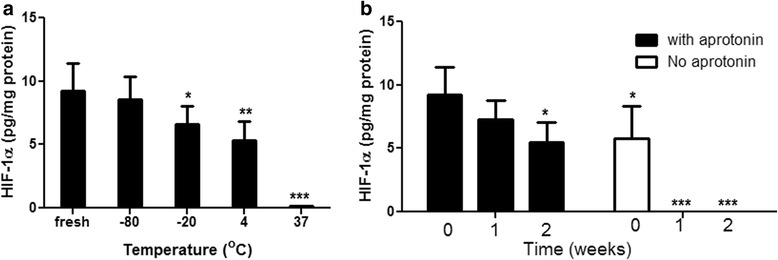
Effect of (**a**) temperature, (**b**) storage time and presence of a protease inhibitor (aprotinin) on the degradation of HIF-1α in cell lysates. The data are mean ± SEM of 3 independent experiments (triplicates in each experiment), *P<0.05, **P < 0.01 and ***P < 0.001 compared with freshly collected samples by ANOVA

Figure 3a shows the detection of HIF-1α by ELISA in cell lysates kept for 24 h at different temperatures. Storage at -80 °C appears essential for the optimal preservation of the protein. In the experiment shown in Fig. 3b, samples were kept at -80 °C for prolonged periods of time, with and without aprotinin. It is very clear that a protease inhibitor is essential for preventing the degradation of HIF-1α in the stored samples. Other protease inhibitors such as Complete Mini protease inhibitor cocktail (Roche) were tested and showed similar protection against HIF-1α degradation (data not shown).

### Measurement of HIF-1α in cell lysates and plasma samples

**Fig. 4:**
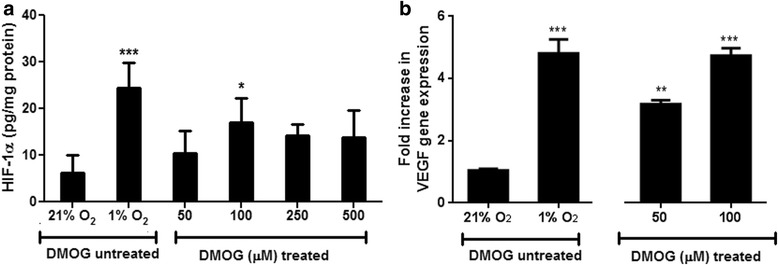
Concentration response curve showing (**a**) HIF-1α levels and (**b**) fold change in VEGF gene expression in cell lysates after treating rat aortic endothelial cells with different concentrations of DMOG (0–500 uM). Cells incubated at 21% O_2_ were used as the untreated control group and cells incubated at 1% O_2_ were used as a positive control for HIF-1α induction. The data are mean ± SEM of 6 independent experiments (triplicates in each experiment) where * *P* < 0.05, **P < 0.01 and ***P < 0.001

HIF-1α was detected in cell lysates treated with DMOG. HIF-1α was measured in cell lysates using the optimized sample collection discussed in the previous section. HIF-1α was induced using DMOG. DMOG caused a significant increase HIF-1α levels when incubated with RAECs for 2 h at a concentration of 100 μM (Fig. 4a) and resulted in transcriptional activation of HIF-1α target genes, increasing the expression of VEGF gene 3–4 fold (Fig. 4b).

### HIF-1α is increased in cell lysate in an in vitro injury model

**Fig. 5:**
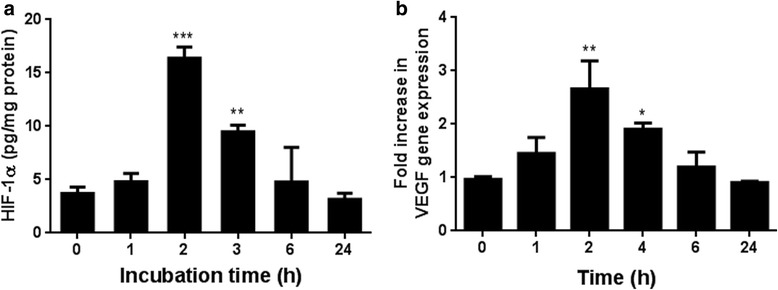
Time course of the levels of intracellular HIF-1α (**a**) and VEGF mRNA (**b**) after a scratch assay in vitro. HIF-1α was measured by ELISA and VEGF mRNA by qPCR. HIF-1α levels are expressed as pg/mg protein and VEGF mRNA levels as arbitrary units versus the uninjured samples. Data are the mean ± SEM of 6 samples. *P < 0.05, P** < 0.01 and ***P < 0.001

The kinetics of HIF-1α release was investigated in the scratch assay model. HIF-1α concentrations in the lysate reached a maximum at approximately 2 h post injury, after which they gradually returned to baseline (Fig. 5a). This pattern of HIF-1α levels was mirrored by changes in VEGF gene expression (Fig. 5b).

### Circulating HIF-1α in rats treated with DMOG

**Fig. 6:**
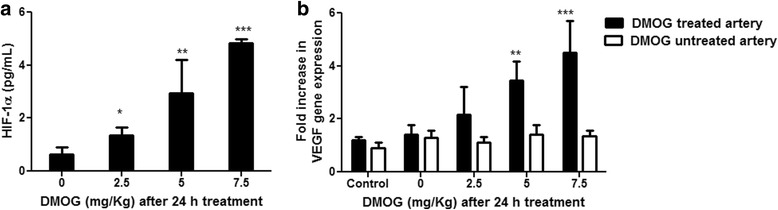
Induction of plasma HIF-1α (**a**) and VEGF gene expression in the carotid artery tissue (**b**) after local application of DMOG (0–7.5 mg/Kg) as arbitrary units versus the uninjured samples. Data are the mean ± SEM (*n* = 6), *P < 0.05, **P < 0.01, ***P < 0.001

HIF-1α was increased in plasma after treatment of the carotid artery with DMOG applied locally at a concentration ranging from 0 and 7.5 mg/Kg in a dose-dependent fashion (Fig. 6a). VEGF gene expression in the treated carotid arteries was consistent with the increased levels of plasma HIF-1α following DMOG administration, but there was no increase in the contralateral, untreated artery (Fig. 6b).

### Circulating HIF-1α after in vivo arterial injury

**Fig. 7:**
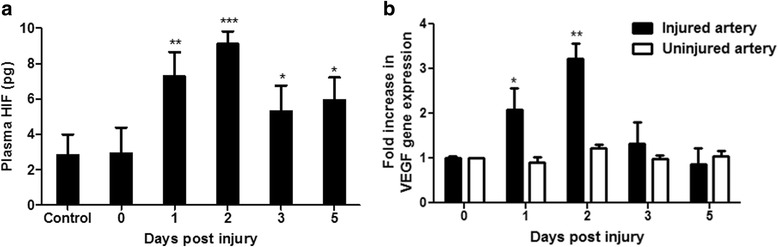
**a** Increase in plasma HIF-1α in a rat angioplasty model. Plasma levels of HIF-1α, measured by ELISA, increased after injury, reaching a maximum after 48 h. (**b**) VEGF mRNA levels in the injured carotid and the contralateral carotid of the same rats as arbitrary units versus the uninjured samples. Data are mean ± SEM (*n* = 6). *P < 0.05, ** *P* < 0.01 and *** *P* < 0.001

The kinetics of HIF-1α release was then measured in a rat model of carotid angiolplasty, using the optimised conditions for plasma sample collection and storage, prior to analysis using the ELISA method. HIF-1α release reached a maximum at approximately 2 days after injury, and then fell over the next 5 days, but remained above baseline (Fig. 7a). In a separate experiment, we looked at the effect of adding proteinase inhibitors to the blood immediately after collection before plasma separation. This has been done by spiking the blood with HIF-1α protein (0.6 ng/mL). Blood samples were then split where protease inhibitor was added to one group after which plasma was separated. In the other group, plasma was separated first prior to the addition of protease inhibitor. Samples were then stored for 48 h prior to analysis. This showed that there was no significant difference between adding the protease inhibitor to the blood immediately after collection or to the plasma after separation and found that this was equally effective. Blood samples were kept on ice after collection and plasma was separated rapidly. Although adding the inhibitor immediately to the blood rather than after plasma separation did not increase the recovery of HIF-1α further (See Additional file 1), this may be more practical in a clinical setting.An increase in local VEGF gene expression in the carotid artery was only observed in injured arteries reaching a maximum level at 2 days after injury, consistent with changes in plasma HIF-1α, and falling to baseline by the fifth day after injury. Uninjured contralateral arteries from the same rats showed no change in VEGF gene expression (Fig. 7b).

### HIF-1α localization

**Fig. 8:**
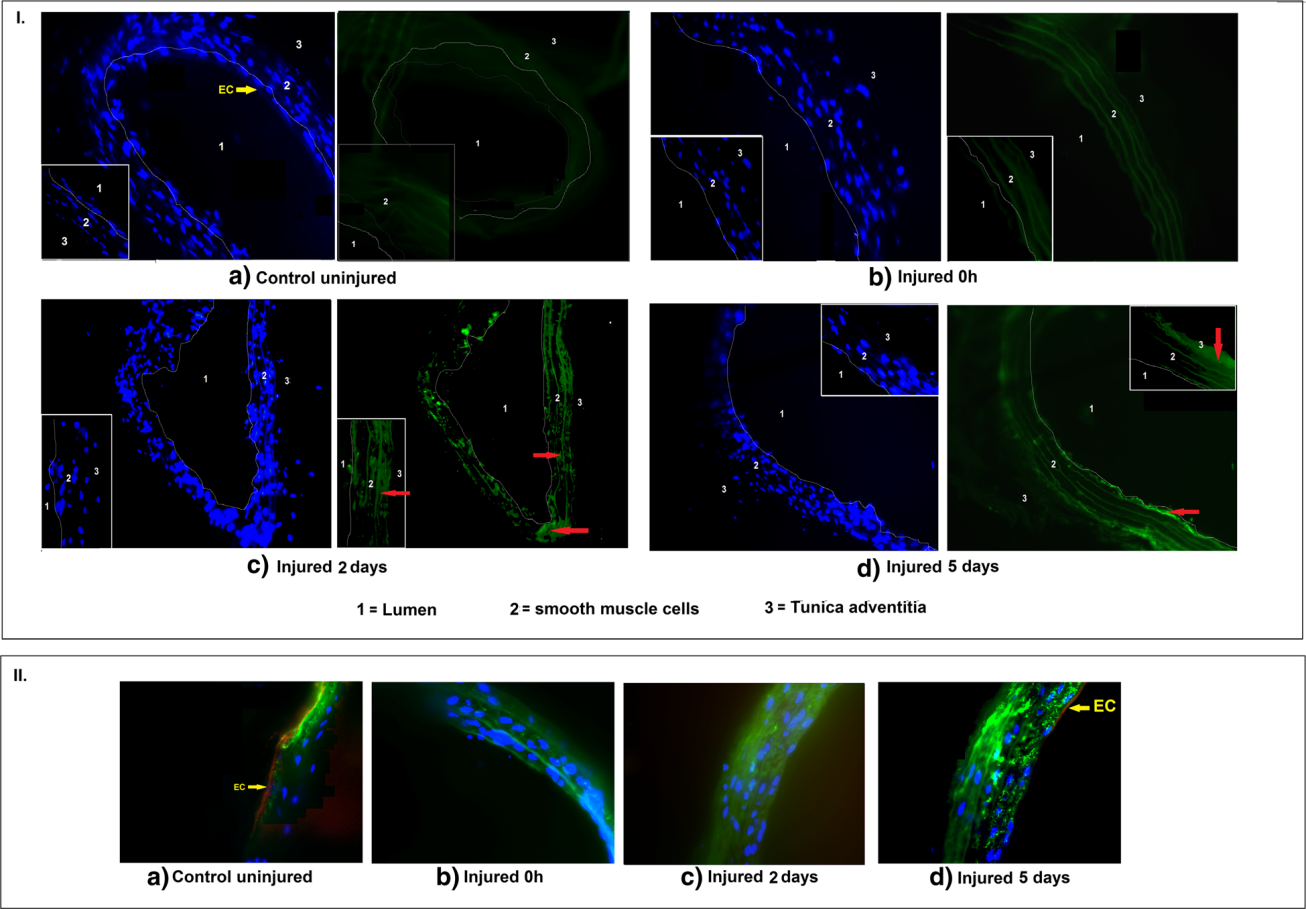
Panel I represents immunohistochemistry of (**a**) untreated carotid arteries and injured carotid arteries (**b**) immediately after injury (**c**) 2 days post injury and (**d**) 5 days post injury using a Leica CTR 5000 fluorescence microscope. Arteries were stained with mouse anti-HIF-1α and followed by FITC-conjugated anti-mouse secondary antibody (green stain) to visualize HIF-1α. Nuclei were stained with DAPI (Blue stain). 1 represents the lumen, 2 represents the smooth muscle cells and 3 represents the Tunica adventitia. Images shown are at 40× magnification and 63× magnification (inset images). Red arrows indicate the localization of HIF-1α expression in the smooth muscle cells. Panel II represents the staining of endothelial cells (EC) and smooth muscle cells (SMCs). The yellow arrow indicates EC layer in control (uninjured) arteries which is removed post injury and starts to regenerate 5 days post injury. Arteries were stained with rabbit anti-CD31 and mouse anti-alpha smooth muscle actin followed by Alexafluor 647 conjugated anti-rabbit secondary antibody (red stain) and FITC-conjugated anti-mouse secondary antibody (green stain) to visualize EC and SMCs respectively

Tissue HIF-1α levels were also assessed in the injured carotid arteries using immunohistochemistry. An increase was observed in the smooth muscle cells that was maximal 2 days after injury (Fig. 8c) and still detectable at 5 days (Fig. 8d). HIF-1α was co-localised in the smooth muscle cells after injury.

## Discussion

A reliable and sensitive HIF-1α ELISA was established and used to assess the stability of HIF-1α in plasma and tissue culture medium. Samples could be stored for several days at -80 °C but required the addition of aprotinin. HIF-1α was released following endothelial injury in vitro and in vivo, in the balloon injury model in the rat.

Hypoxia has important effects on intermediary metabolism, cholesterol disposal, and the inflammatory response, and promotes changes in the cellular and extracellular composition of the artery wall that may impact on the response to injury and atherosclerosis. HIF and its down-stream effector gene products have been increasingly recognized for their key role in regulating a wide-spectrum of cellular events, including angiogenesis, cell proliferation, apoptosis and protective response to limit tissue damage (Imtiyaz and Simon 2010; Walshe and D'Amore 2008). There is also increasing evidence that HIF-1α plays a critical role in an early cardioprotective response (Ke and Costa 2006; Tekin et al. 2010), whilst HIF-2α mediates a delayed cardio protective effect, playing an important role in adapting the cells to chronic hypoxia and injury (Bautista et al. 2009; Ong and Hausenloy 2012). Therefore measurement of HIF-1α rather than HIF-2α may be useful for the assessment of acute disease severity and prognosis.

### The measurement of HIF-1α in plasma and cells

Although HIF-1α is known to be unstable, there have been previous reports of the measurement of HIF-1α in plasma in man and in rats (He et al. 2016b; Li et al. 2015; Xu et al. 2016). However the description of the sample collection and storage procedures are poorly reported in these studies. In this current paper we have confirmed that HIF-1α is indeed unstable, and that careful collection procedures are essential to prevent its degradation. We found that blood samples and cell lysates can be stored for up to two weeks at -80 °C in the presence of protease inhibitors. Whilst we used aprotinin at a final concentration of 300 nM, we found that other protease inhibitors such as Complete Mini protease inhibitor cocktail tablets (Roche) have a similar effect (data not shown).

The HIF-1α ELISA was robust, of adequate sensitivity and precision for use in biological samples. The values obtained using the ELISA were consistent with Western blot analysis using different HIF-1α antibodies. The measurement of HIF-1α in the in vitro studies was also consistent with expectations: hypoxia and a known HIF-1α inducer increased intracellular HIF-1α levels and down-stream VEGF expression. HIF-1α is involved in regulating the expression of VEGF, which is one of the most potent proangiogenic growth factors (Song et al. 2017). The expression of VEGF was measured to confirm that the levels of HIF-1α in the biological samples were sufficient to cause down-stream signalling.

We observed that there was a transient increase in the expression of HIF-1α and VEGF in a model of rat aortic endothelial cell (RAECs) injury in vitro. The rapid increase in HIF-1α levels from injured RAECs reached a peak level at approximately 2 h post injury in the scratch model. This occurred under normoxic conditions and in the absence of inflammatory cells showing that HIF-1α is directly involved in the adaptive response to cell injury.

### HIF-1α and vascular injury

vIn vivo, DMOG, applied locally to a rat carotid artery, caused a systemic increase in plasma HIF-1α that peaked at 2 days, and was approximately 3-fold higher than baseline values. This was associated with an up-regulation of the local, but not the contralateral VEGF arterial tissue expression. HIF-1α appears to be an important mediator of the pro-atherogenic cellular response to hypoxia and is up-regulated in inflammatory and hypoxic areas of carotid arteries in simple and complex lesions from patients with carotid artery disease. In vascular smooth muscle cells, HIF-1α can be activated under normoxic conditions by platelet products such as platelet derived growth factor and thrombin (Gorlach et al. 2001). In the rat carotid balloon injury model, platelets are deposited on the injured artery within minutes of injury, and that they are important in the subsequent formation of the neo-intima (Fingerle et al. 1989). In a murine model of vascular injury, HIF-1α expression was shown to be increased in the smooth muscle cells of the tunica media, as early as 1 day after injury, HIF-1α mRNA expression was induced at 6 h after injury, and its inhibition by the local application of HIF-1α -siRNA reduced neo-intimal area by 49% and significantly decreased the neo-intimal smooth muscle cells content. HIF-1α expression therefore appears to be directly involved in the formation of the neo-intima after vascular injury (Karshovska et al. 2007).

## Conclusions

It is possible to measure HIF-1α in biological samples using an ELISA assay, although careful sample preparation and storage is essential. This may be of value in conditions in which tissue hypoxia may play a role, including cancer, cardiovascular disease and pulmonary disease.

HIF-1α is rapidly up-regulated in an in vitro*,* wound-healing model, the scratch assay, even in the absence of hypoxia. It is therefore likely to play an important role in orchestrating the response to injury.

The up-regulation of HIF-1α expression and release was more sustained in the rat balloon injury model, where plasma HIF-1α reached its peak concentrations 2 days post injury. The differences in the kinetics of HIF-1α expression and release for the in vitro and in vivo models could be due to the complexity of the in vivo model. The latter model involves several cell types, including VSMS, platelets and inflammatory cells; these may interact to stimulate pathways that could lead to a more sustained HIF release that may have implications for conditions such as coronary atherosclerosis, restenosis and stroke.

## Additional file

Additional file 1: **Figure S1**. Effect of adding the protease inhibitor; aprotinin on HIF-1α levels in plasma when added directly to the blood after collection versus addition to the plasma after separation. Blood was spiked with HIF-1α protein (0.6 ng/mL). Blood samples were then split into two groups where the  protease inhibitor was added to one group after which plasma was separated. In the other group, plasma was separated first prior to the addition of protease inhibitor. Data represented are the mean ± SEM of 3 rats (triplicate of each). (DOCX 23 kb)

## Abbreviations

DMOG: Dimethyloxalylglycine
ELISA: Enzyme linked immunosorbent assay
HIF-1α: Hypoxia inducible factor-1 alpha
RAECs: Rat aortic endothelial cells
VEGF: Vascular endothelial growth factor
