# Correction to: Assessment of HIF-1α expression and release following endothelial injury in-vitro and in-vivo

**DOI:** 10.1186/s10020-020-00188-w

**Published:** 2020-06-25

**Authors:** Lamia Heikal, Pietro Ghezzi, Manuela Mengozzi, Gordon Ferns

**Affiliations:** 1 Brighton and Sussex Medical School Department of Clinical and Experimental Investigation, University of Sussex, Falmer East Sussex, Brighton, BN1 9PS UK; 2 Brighton and Sussex Medical School Department of Medical Education, Mayfield House, Falmer East Sussex, Brighton, BN1 9PH UK; 3 Department of Pharmaceutics, Faculty of Pharmacy, Alexandria University, Alexandria, 21521 Egypt

**Correction to: Mol Med (2018) 24:22**

**https://doi.org/10.1186/s10020-018-0026-5**

Following publication of the original article (Heikal et al. 2018), the author Lamia Heikal would like to update her affiliation by adding a new one. The updated author affiliation is shown in this erratum.

Correct:

^1^Brighton and Sussex Medical School Department of Clinical and experimental investigation, University of Sussex, Falmer East Sussex, Brighton BN1 9PS, UK

^3^Department of Pharmaceutics, Faculty of Pharmacy, Alexandria University, Alexandria, 21521, Egypt
