# Supplementary material for: Assessment of HIF-1α expression and release following endothelial injury in-vitro and in-vivo
Source: Mol Med. 2018 May 16;24:22. doi: 10.1186/s10020-018-0026-5 (PMC6016879; doi:10.1186/s10020-018-0026-5)
Supplement: Supplementary file 1 — Figure S1. Effect of adding the protease inhibitor; aprotinin on HIF-1α levels in plasma when added directly to the blood after collection versus addition to the plasma after separation. Blood was spiked with HIF-1α protein (0.6 ng/mL). Blood samples were then split into two groups where the protease inhibitor was added to one group after which plasma was separated. In the other group, plasma was separated first prior to the addition of protease inhibitor. Data represented are the mean ± SEM of 3 rats (triplicate of each). (DOCX 23 kb) [file 10020_2018_26_MOESM1_ESM.docx]

Additional file 1:


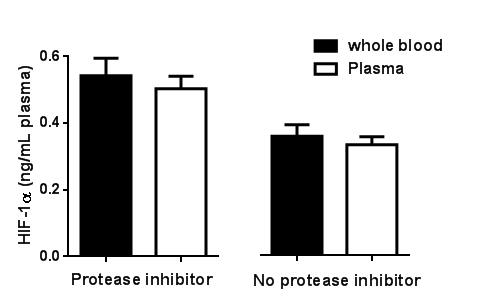


Figure 1: Effect of adding the protease inhibitor; aprotinin on HIF-1α levels in plasma directly to the blood after collection versus addition to the plasma after separation. Blood was spiked with HIF-1α protein (0.6 ng/mL). Blood samples were then split where p protease inhibitor was added to one group after which plasma was separated. In the other group, plasma was separated first prior to the addition of protease inhibitor. Data represented are the mean ± SEM of 3 rats (triplicate of each).
